# Detection of Unknown Pregnancy With Complications Using Point-of-Care Ultrasound

**DOI:** 10.7759/cureus.16510

**Published:** 2021-07-20

**Authors:** Allison N Kayne, Julie A Fritzges, Michelle L Huang, Elizabeth Evans

**Affiliations:** 1 Department of Emergency and Hospital Medicine, Lehigh Valley Health Network, Allentown, USA; 2 Department of Obstetrics and Gynecology, Lehigh Valley Health Network, Allentown, USA

**Keywords:** eclampsia, unknown pregnancy, point-of-care ultrasound, pregnancy complications

## Abstract

Eclampsia, a condition diagnosed in pre-eclamptic patients who experience seizures, can lead to maternal and fetal death if not treated early. The present case discusses the clinical management of an 18-year-old female who presented to the emergency department (ED) after a generalized tonic-clonic seizure. A physical examination revealed that she was also hypertensive. Based on these symptoms which required urgency due to the patient’s instability, and the suspicion that the patient could be pregnant, point-of-care ultrasound (POCUS) was performed. In this case, a POCUS was a faster more accessible modality than a urine or serum human chorionic gonadotropin test. Although the patient denied that she was pregnant, POCUS identified that she was approximately 22-24 weeks pregnant. The patient was promptly diagnosed with eclampsia and given medication to control her blood pressure and seizures. This case highlights the benefits of using POCUS in the ED to expedite clinical decisions by identifying the etiology of a patient’s condition and lends itself to the discussion of its utility in a critically ill pregnant woman. It also serves to reinforce the importance of keeping eclampsia as part of an emergency physician’s differential when confronted with a potentially pregnant patient with relevant symptoms.

## Introduction

Approximately 14% of all maternal deaths globally are a result of hypertensive disorders during pregnancy, of which the majority of mortalities are associated with pre-eclampsia and eclampsia [1]. Pre-eclampsia is characterized by an onset of hypertension (systolic blood pressure of ≥140 mmHg and/or diastolic blood pressure of ≥90 mmHg) after 20 weeks of gestational age and up to 6 weeks postpartum with either end-organ dysfunction or proteinuria [2]. This disorder affects the remodeling of uterine arteries, known as spiral arteries, which are important for uterine perfusion during early pregnancy [3]. In a healthy patient, the spiral arteries are remodeled from high-resistance vessels into larger, dilated vessels with reduced blood pressure [3]. In patients with pre-eclampsia, this remodeling is impaired; as such, the spiral arteries remain narrow which can lead to defective placentation, placental hypoperfusion, and eventually the symptoms of pre-eclampsia [4]. Adverse conditions of pre-eclampsia can affect multiple organ systems and lead to severe complications, including eclampsia, which is characterized by the additional development of seizures [2]. Eclampsia, if untreated, can be life-threatening; thus, it is imperative for this condition to be recognized early and treated promptly.

The present case discusses the clinical management of an 18-year-old female who presented to the emergency department (ED) after a generalized tonic-clonic seizure. The use of point-of-care ultrasound (POCUS) in the ED rapidly identified the etiology of the patient’s clinical presentation, an unknown pregnancy. Detection of this unknown pregnancy using POCUS directed clinical management of our patient and expedited her treatment for eclampsia. This case also lends itself to further discussion of the utilization of POCUS in critically ill pregnant patients.

## Case presentation

**Figure 1:**
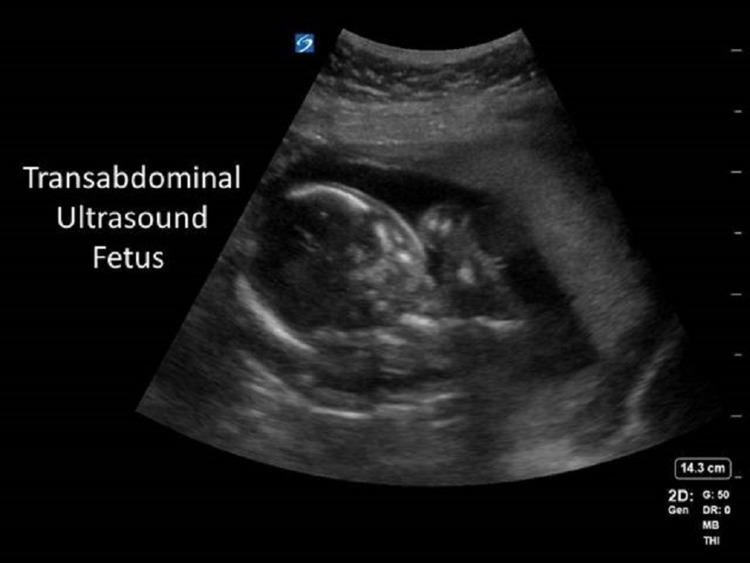
Representative transabdominal ultrasound fetus.

**Figure 2:**
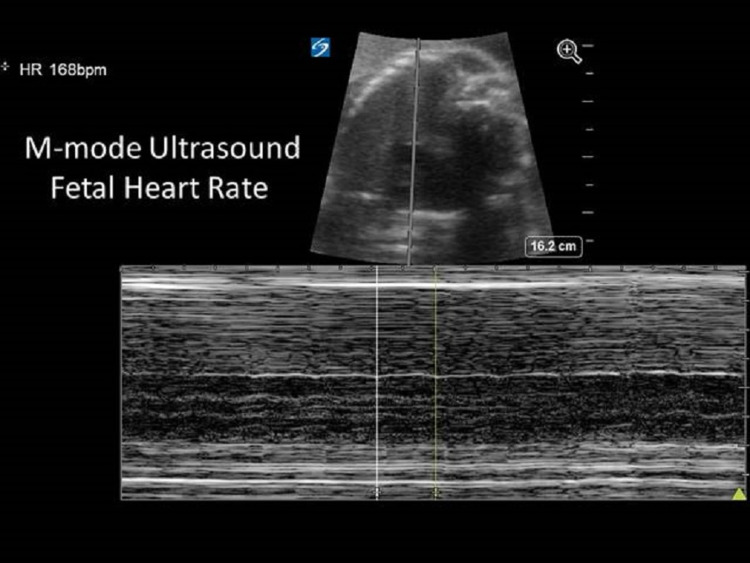
Representative M-mode ultrasound showing fetal heart rate.

An 18-year-old female with a history of asthma presented to the ED after a generalized tonic-clonic seizure with loss of consciousness. The patient’s family reported that she had been complaining about a migraine, swelling in her feet, and blurred vision for the past couple of days. In the ED, the patient seized again, so a noncontrast computed tomography (CT) scan of her head was ordered, and she was given 2 mg lorazepam (Ativan). Her vital signs were as follows: blood pressure of 175/115 mmHg, pulse rate of 111 beats per minute, respiratory rate of 28 breaths per minute, temperature of 36.7°C (98.1°F), and oxygen saturation of 100%. A bedside ultrasound was performed to check for the possibility of pregnancy due to the patient’s reported symptoms, although the patient had denied the possibility of being pregnant. Unbeknownst to the patient and her family, the patient was pregnant; the ultrasound positively identified an intrauterine pregnancy and it was estimated that the patient was 22-24 weeks pregnant (Figures 1, 2).

The uterine fundus was palpated approximately 2-4 cm above the umbilicus and a fetal heart rate was detected at 168 beats per minute. The patient was given a bolus of 4 g of magnesium sulfate and started on a continuous infusion. In addition, she was given 20 mg of intravenous (IV) labetalol to control her blood pressure.

**Figure 3:**
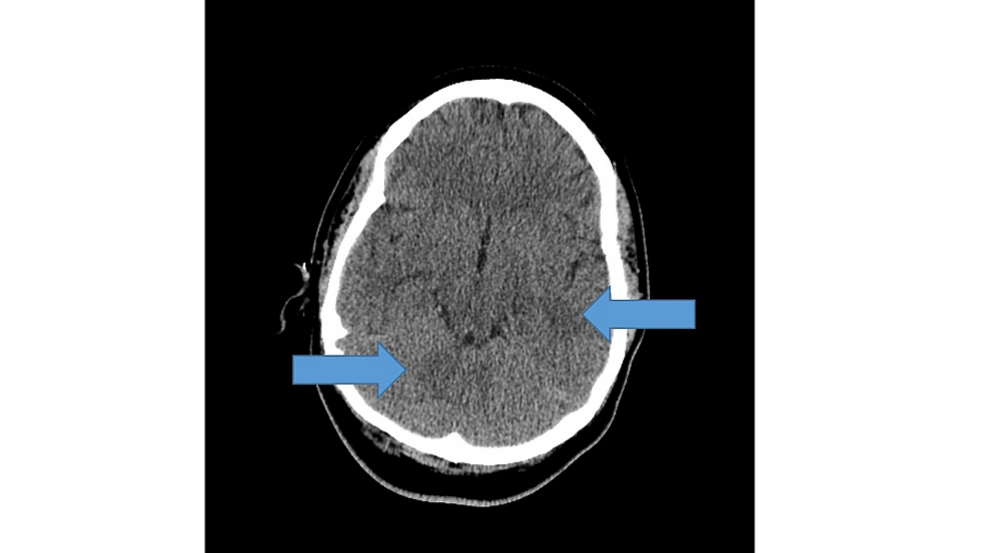
Computed tomography of the head showing subtle patchy hypodensities (arrows) seen in the basal ganglia as well as the parietal, occipital, and frontal lobes.

The patient’s labs revealed the following: hemoglobin 15.7 g/dL (normal: 11.5-14.5 g/dL), white blood cell count 25,300/mm^3^ (normal: 4,000-10,000/mm^3^), platelet count 210,000/mm^3^ (normal: 140,000-350,000/mm^3^), creatinine 1.17 mg/dL (normal: 0.40-1.10 mg/dL), alkaline phosphatase 177 U/L (normal: 35-120 U/L), alanine aminotransferase 76 U/L (normal: <56 U/L), aspartate aminotransferase 78 U/L (normal: <41 UL), and proteinuria. The results of the patient’s head CT scan showed abnormal hypodense lesions in the frontal lobes, basal ganglia, parietal, and occipital lobes, concerning for infarcts or septic emboli (Figure 3).

The patient was promptly diagnosed with eclampsia with acute kidney injury. Because the ED department where the patient presented is a small, urban ED without in-house admission capabilities, she was transferred via ambulance to the labor and delivery unit at the hospital network’s tertiary care campus.

Upon arrival to labor and delivery, the patient’s blood pressure continued to be in a severe range. She required an additional 20, 40, and 80 mg of IV labetalol and 10 mg of IV hydralazine with minimal response. Another bedside ultrasound was performed and revealed a fetal weight of 919 g (2 pounds), an amniotic fluid index of 7.9 cm, and cephalic presentation. No obvious malformations were seen. Fetal breathing, hiccups, and movement were also found, giving the baby a biophysical profile of 6/8 due to low fluid. The patient was sent to get a magnetic resonance imaging to further image her brain, but began to contract more frequently with recurrent late decelerations, prompting an immediate C-section. The baby was born weighing 825 g (1 pound, 13.1 ounces) and estimated to be 26 weeks old.

The patient remained intubated and sedated after delivering her baby and was transferred to the Neuroscience Intensive Care Unit for ongoing management. She was discharged a week later with an antihypertensive regimen, Nexplanon® for contraception, and follow-up appointments to an OB/GYN. Her daughter remained under intensive care for ongoing thermoregulatory and nutritional management.

## Discussion

To our knowledge, this is one of only a few cases that illustrate the use of POCUS in facilitating the diagnosis of eclampsia in a patient who did not know she was pregnant. POCUS has become a widely used diagnostic tool in the ED [5,6]. This type of imaging has become especially important for patients with life-threatening conditions necessitating immediate diagnoses, such as those with pericardial tamponade and ectopic pregnancies [7,8]. However, there has been controversy over whether there are benefits in emergency clinicians using POCUS to evaluate conditions that are not time-sensitive, such as first-trimester pregnancy complications or gallbladder issues [9].

Despite this controversy, POCUS has been evaluated for its efficacy in safely and accurately diagnosing intrauterine pregnancies. Several studies have shown that POCUS not only accurately identifies intrauterine pregnancies but also reduces patient length of stay [9,10]. POCUS has also been used to identify complications associated with pregnancy. A previous report showed the importance of ocular POCUS in identifying bilateral retinal detachments indicative of pre-eclampsia in a female patient who did not know she was pregnant [11]. The present case adds to the existing literature by confirming the benefits of using POCUS to quickly identify intrauterine pregnancies to facilitate a patient’s clinical management.

Identifying this condition in our patient was not only important for her health but also for her new baby, as infants of mothers with pre-eclampsia can experience severe health consequences. Neonatal mortality is approximately two times higher among infants of mothers with pre-eclampsia, and the infants have an increased risk of febrile seizures, low Apgar scores, and neonatal intensive care unit admission [12]. Exposure to pre-eclampsia in-utero has also been shown to have long-term health effects on infants, including higher levels of systolic and diastolic blood pressure during childhood, increased risk of metabolic disorders, and higher risk for epilepsy and stroke [12].

In this case, the patient presented to the ED after having a seizure and was unstable with a broad differential on arrival and a reported denial of pregnancy. The POCUS helped narrow the differential rapidly and facilitate immediate treatment. There has been support for POCUS examination of the lungs, heart, and optic nerve sheath diameter to help detect the presence of pulmonary interstitial edema, cerebral edema, and/or cardiac dysfunction while managing patients with pre-eclampsia and eclampsia [13]. Some managing critically ill pregnant women suggest opportunities for further use of multiorgan POCUS including transcranial Doppler and transcranial color-coded duplex sonography which are not traditionally done in the ED setting [14]. While it was not performed in our case, optic nerve sheath diameter to assess for intracranial pressure can be considered in critically ill pregnant women [14]. While other specialties such as critical care and anesthesiology may have more experience as a specialty using POCUS in critically ill pregnant patients, in our case the modality was used to reach the diagnosis not to guide the treatment which ultimately was received at a definitive care facility. In the short term, POCUS enabled a swift diagnosis so the patient could be started on blood pressure medication and receive magnesium sulfate, which has been found to significantly reduce the adverse outcomes associated with this condition [15], to control her symptoms and the progression of her condition. In addition, the fetus could be monitored and an informed decision could be made about the urgency of delivery.

## Conclusions

This case shows the benefits of using a quick imaging technique such as POCUS to identify the etiology of a patient’s condition, which in our case was an intrauterine pregnancy. Discovering the patient’s unknown pregnancy using POCUS was instrumental in providing both the patient and her baby with the urgent care they needed. In patients of childbearing age with an unreported pregnancy who present with the described symptoms of eclampsia, we recommend that emergency physicians incorporate the timely use of POCUS to assess for the possibility of pregnancy to expedite diagnosis and treatment. More advanced POCUS imaging holds promise for further management of critically ill pregnant patients. In addition, although eclampsia is rare, we suggest that emergency physicians keep this condition as part of their differential when evaluating potentially pregnant patients with relevant symptoms.
